# Computational Perspectives on Amoxicillin and Staphylococcus Aureus in Mirror Life

**DOI:** 10.1002/gch2.202500051

**Published:** 2025-07-02

**Authors:** Lorenzo Pedroni, Chiara Dall'Asta, Gianni Galaverna, Luca Dellafiora

**Affiliations:** ^1^ Department of Food and Drug University of Parma Parco Area delle Scienze 27/A Parma 43124 Italy

**Keywords:** antibiotics, computer simulations, environmental safety, mirror life, staphylococcus aureus

## Abstract

The concept of mirror life is first introduced by Louis Pasteur, referring to biological systems composed of enantiomeric biomolecules. Although nowadays technologies are making a mirror life theoretically achievable, its potential risks remain uncertain. Here, an integrated multi‐tier computational pipeline is employed to address the potential environmental threat posed by the hypothetical mirror‐image of *Staphylococcus aureus*, a bacterium relevant to environmental and food safety. The findings suggest that amoxicillin, and perhaps other conventional antibiotics, should not be effective against their mirror targets. On the other hand, the enantiomeric amoxicillin may be a successful counteracting measure, although the risks for the biosphere remain unknown. Overall, this study highlights the need for further dedicated investigations in this field, while emphasizing *in silico* methods, in particular molecular modeling, as a versatile and effective first‐line approach for analysis, free from biohazards and technical limitations of reagents supply.

## Graphical abstract

Mirror life is a possible incoming threat. PBP3 of *S. aureus*, a well‐known target of amoxicillin and other β‐lactam antibiotics, is investigated along with its mirror image showing that the enantiomeric target may elude currently available biocides. Computational tools are safe and reliable to investigate mirror‐life related aspects, either supporting the development of countermeasures or driving the assessment of risks.

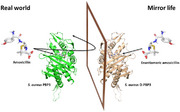

## 1 Introduction

In the middle of the 1800s, Louis Pasteur was a pioneer in opening the debate around mirror life, including discussions about enantiomeric versions of its building blocks like left‐handed carbohydrates and nucleotides, and right‐handed proteins and amino acids. During the same period, in a milestone of nineteenth‐century literature (*Through the Looking‐Glass, and What Alice Found There*), Alice wondered if the glass of milk she could see through the looking‐glass was good to drink. Since then, scientists and novelists speculated, romanticized, and debated around that topic. Nowadays, the term “mirror life” refers to biological systems either substantially or purely made of enantiomeric counterparts replacing those naturally selected as Life's building blocks in the biosphere.^[^ ^1^, ^2^ ^]^ Recently, concrete steps toward the creation of “mirror life” have been moved thanks to technological advances like the development of mirror‐image nucleic acid polymerases and the theorization of enantiomeric ribosomes.^[^ ^2^, ^3^ ^]^

Today, mirror life organisms are still theoretical, but the body of knowledge accumulated to date should restrain the Scientific Community from taking steps forward without first developing effective containment strategies. The prompt development of effective countermeasures is even more important considering the possible overcoming of technological limitations that still hamper the creation of mirror microorganisms in the near future. Recently, in a Policy Forum published in *Science*, Adamala et al., warned that mirror bacteria may pose an unprecedented menace to Life for several reasons, such as the likely evasion of immune systems and the risk of a strikingly enhanced fitness and competitiveness.^[^ ^4^ ^]^ The authors have also encouraged broadening the discussion on the topic to uncover all the possible associated issues.

Though the risks mirror bacteria may pose to the environment and living organisms are still debated (see the *Science* eLetter “*In response to “Confronting risks of mirror life*”” by Prof. Perrin as a response to the paper of Adamala et al.), there is consensus that it is necessary to carefully assess the risks before enabling their creation. However, the assessment of risks is hampered by technical impairments, like those limiting the production of D‐proteins and other mirror (macro)molecules, but computational modeling may reliably overcome these limits.

With this respect, we created an open forum for cumulative research and discussion on the risks associated with mirror microorganisms^[^ ^5^ ^]^ where we stated the importance of computational modeling to provide a safe means to pre‐test and design strategies to tackle this threat (see our *Science* eLetter “*Antibiotics Through the Looking‐Glass: Computational Approaches to Tackling the Threats of Mirror Life*” as a response to the paper of Adamala et al.).

**Figure 1:**
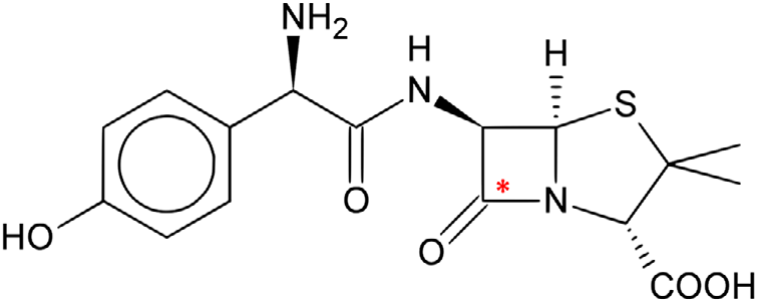
2D structure of amoxicillin (PubChem CID 33613). The red asterisk indicates the reactive carbon (carbonyl carbon of the β‐lactam ring) forming the covalent bond with the Ser392 of the investigated PBP3.

In this paper, we considered a hypothetical mirror image of *Staphylococcus aureus*, a widespread environmental bacterium responsible for severe foodborne human infections,^[^ ^6^ ^]^ which may raise significant concerns in terms of environmental safety and hygiene. As a proof‐of‐principle, we applied a previously validated computational workflow (e.g., ref. [5, 7, 8]) to test whether amoxicillin (**Figure** **1**) could effectively target this bacterium in a mirror life scenario. Amoxicillin is known to be effective against β‐lactams‐sensitive strains of *S. aureus* ^[^ ^9^ ^]^ by binding to the transpeptidase (TP) domain of the Penicillin Binding Protein 3 (PBP3).^[^ ^10^, ^11^, ^12^, ^13^, ^14^ ^]^ We investigated whether amoxicillin would similarly bind to the mirror image of this *S. aureus* target protein.

## 2 Results and Discussion

The computational workflow applied here integrated molecular docking with MD simulations as previously succeeded to predict the stability of protein‐ligand complex on a variety of systems (e.g., ref. [7, 8]). In this study, we analysed whether amoxicillin, which is effective against not methicillin‐resistant strains of *S. aureus*,^[^ ^9^ ^]^ could also be effective against the mirror image of *S. aureus* PBP3, a known target of β‐lactams antibiotics.^[^ ^10^, ^11^, ^12^, ^13^, ^14^ ^]^ A fit‐for‐purpose validation was performed to verify the case‐specific reliability of the procedure applied by studying the overall geometrical stability of the apo models, subsequently calculating the interaction between amoxicillin and its target.

**Figure 2:**
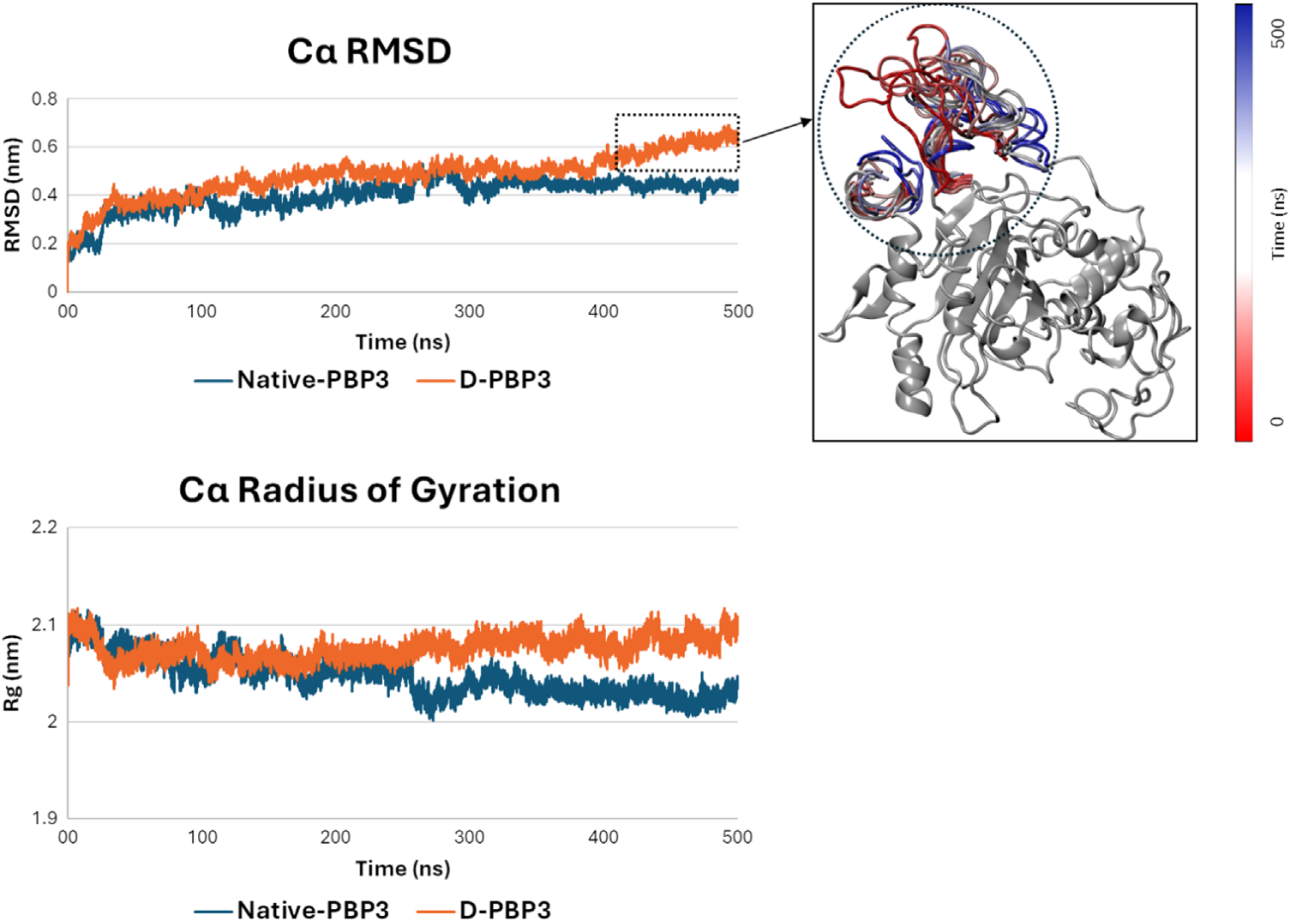
MD results of the native and D‐PBP3 unbound state. The Root Mean Squared Deviation (RMSD) of protein carbon alpha (Cα) plot for native and D‐PBP3 is reported on top. In the right close‐up the loop trajectory is shown in the time‐step representation (red to blue – 0 to 500 ns; protein core is represented as single frame for better clarity). The dotted ring indicates the region responsible for the slight RMSD increase of the D‐PBP3 from 400 ns onward. On the bottom, it is reported the radius of gyration (Rg) plot calculated on protein Cα for native and D‐PBP3.

As shown in **Figure** **2**, both apo models showed geometrical stability (expressed as RMSD and gyration radius trends) over the timeframe considered, ultimately validating models' reliability. Both systems were geometrically equilibrated from ≈150 ns onward, though in one of the two D‐PBP3 replicas, a slight increase (≈0.1 nm) occurred ≈400 ns. A close inspection of the trajectory revealed this was due to a local rearrangement of a side region of the protein, which is involved in protein‐protein interaction and not relevant for amoxicillin binding (Figure 2). However, as a further tier of analysis, the radius of gyration, which reflects the overall conformational equilibrium of the system,^[^ ^15^ ^]^ was calculated for all the replicas. Overall, the two systems behaved alike with similar steady state trends – differences among replicas were below 0.1 nm, underlying the geometrical stability of both.

**Figure 3:**
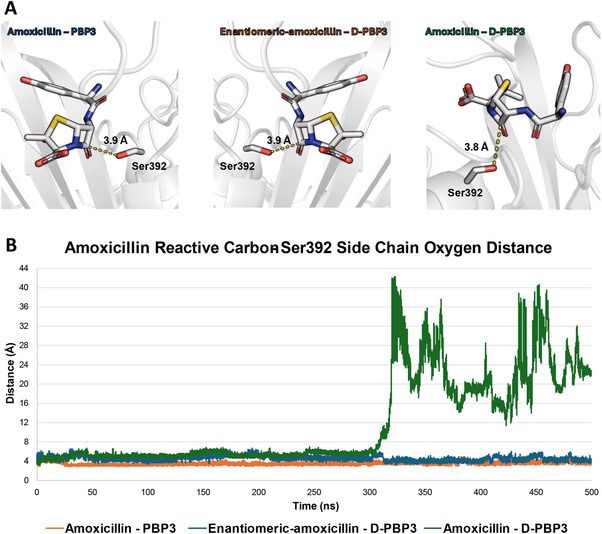
Docking and MD results of the native amoxicillin – P‐PBP3, enantiomeric amoxicillin – D‐PBP3, and amoxicillin – D‐PBP3 complexes. A) Docking results. The protein is shown in cartoon while the amoxicillin and the side chain of Ser392 meant to form the covalent bound with the β‐lactam ring are shown in sticks. The dashed yellow lines indicate the interatomic distance. B) MD results. The plot represents the average distance (Å) over the two replicas between the amoxicillin β‐lactam ring's carbonyl carbon and the Ser392 side chain's oxygen in the complexes under investigation.

Regarding the amoxicillin‐PBP3 complex, the mechanism of action of amoxicillin involves the covalent binding of Ser392 (numbering as per UniProt assignment) through the reactivity of its β‐lactam ring (Figure 1). Aligning with previous studies, the present work studied the persistence of amoxicillin β‐lactam ring close to the side chain of Ser392 to predict its reactivity to form a covalent bond.^[^ ^16^, ^17^ ^]^ The docked pose scored 42 PLPScore units (positive scores indicate the physico‐chemical match between the protein pocket and the docked ligand, as per manufacturer declaration; https://www.ccdc.cam.ac.uk) with the carbonyl carbon arranged close to the Ser392 side chain's oxygen (**Figure** **3**). Also, as shown in Figure 3, the interatomic distance between the Ser392 side chain's oxygen and the amoxicillin carbonyl's carbon was kept stable during the whole simulation. This ultimately validated the model reliability, confirming its capability to model the amoxicillin interaction and subsequent activity.

Concerning the interaction of amoxicillin with the D‐PBP3, the docked score was comparable to that recorded within the native target (44 and 42 units, respectively) as well as the interatomic distances (3.9 and 3.8 Å, respectively). However, MD simulations revealed that the interaction of amoxicillin was not stable within D‐PBP3, showing a variable RMSD trend and wider interatomic distance (11.81 Å as average; Figure 3), which is reasonably not compliant with the possibility to form a covalent bond. Moreover, in one of the two independent MD replicas, amoxicillin completely detached from D‐PBP3. Therefore, though amoxicillin could theoretically interact with D‐PBP3, it was calculated to be neither binding nor inhibiting it efficiently and likely detaching from the binding site. Of note, besides PBP3, other amoxicillin targets exist in *S. aureus* (e.g., PBP2).^[^ ^11^ ^]^ A similar behaviour cannot be excluded for other enantiomeric targets of β‐lactam antibiotics, including amoxicillin. This raises the question whether an enantiomeric *S. aureus* might be inherently resistant to the array of β‐lactams available today.

Based on the hypothesis that a mirror image of amoxicillin could be effective against mirror targets, the enantiomeric‐amoxicillin – D‐PBP3 mirror complex was investigated as well. As expected, it behaved as the native counterpart (Figure 3), aligning with the hypothesis that enantiomeric antibiotics might be effective in the mirror life scenario. Of note, mirror antibiotics are expected to be enantiomer‐specific, thus ineffective versus the L‐systems. In line with this interpretation, which will, however, require dedicated investigations, the development of enantiomeric biocides may ensure improved selectivity against mirror bacteria. If confirmed, this feature would bring benefits such as reduced risk of targeting off‐target species and associated increase in antibiotic resistance. At the same time, this would allow the re‐purposing of biocides that have been discharged due to their toxic profile, considering that certain enantiomers may be way less toxic to living organisms (e.g., ref. [18, 19]). However, the opposite scenario should be considered as well, underlining the need to carefully assess case‐by‐case the safety of enantiomeric antibacterial compounds to avoid the release of biocides with a broad‐spectrum toxicity into the biosphere.

## 3 Conclusion

To the best of our knowledge, this is the first evidence highlighting the intrinsic refractoriness of mirror‐image targets to known biocides. This scenario highlights the need for further dedicated investigations in this field, while establishing *in silico* methods as a versatile and effective first‐line framework for analysis, free from biohazards and technical limitations of reagents supply.

From an environmental perspective, the risk of spreading mirror bacteria could be catastrophic, although still remote. The reason we chose *S. aureus* as a case study is mainly due to its role as a human and animal pathogen and its persistence within several ecosystems, including soil, water, and the food chain.

Importantly, the environment would be the first line of control for implementing effective containment strategies against mirror bacteria. In this scenario, computational modeling may provide a safe space to thoroughly investigate and outline possible containment strategies, avoiding the risk of creating a dangerously unbalanced risk‐benefit scenario.

## 4 Experimental Section

### Data Retrieval

The 3D structure of the PBP3 TP domain (from here on referred to as PBP3) from *S. aureus* was generated through ColabFold (v. 1.5.5), an AlphaFold2‐based notebook,^[^ ^20^ ^]^ starting from the primary sequence stored in the UniProt AC A0A1K9IMW8 (https://www.uniprot.org/).^[^ ^21^ ^]^ Only the domain described in crystallographic studies^[^ ^14^ ^]^ to bind β‐lactam antibiotics (from Gln314 to Gln679 according to the UniProt assignment) was considered for this study. The 3D structure of amoxicillin (CID 33613) was downloaded in the SDF format from PubChem (https://pubchem.ncbi.nlm.nih.gov/).^[^ ^22^ ^]^ It was converted to the MOL2 format in a correct physiological protonation state using Open Babel^[^ ^23^ ^]^ setting the flag –*p 7*. The mirror‐counterpart of both PBP3 and amoxicillin –, i.e., D‐PBP3 and enantiomeric amoxicillin – were generated using an *ad hoc* Python script available on Zenodo,^[^ ^5^ ^]^ which ultimately reversed the sign of each X, Y, and Z coordinate, providing the precise mirror imagine of the complex.

### Molecular Docking

Docking simulations were performed using GOLD (Genetic Optimization for Ligand Docking; v. 2022) software. The internal PLP scoring function was used, providing output scores proportional to the fitting of the ligand within the binding pocket (the higher the score the better the physicochemical match – as per manufacturer declarations https://www.ccdc.cam.ac.uk). The binding pocket's centroid for PBP3 was retrieved by aligning the structure with the Protein Data Bank (PDB) (https://www.rcsb.org/)^[^ ^24^ ^]^ structure having ID 3VSK.^[^ ^14^ ^]^ Regarding D‐PBP3, the centroid was set keeping the same absolute values but with inverted signs. The systems analysed were amoxicillin – PBP3, enantiomeric‐amoxicillin – D‐PBP3, and amoxicillin – D‐PBP3. All the docking protocols were set to favour the formation of a hydrogen bond between the carbonyl group of the amoxicillin's β‐lactam ring and the PBP3's serine (Ser392) involved in the covalent bond.

### Molecular Dynamics Simulations

The docking results were given as input for the following molecular dynamics simulations. These were performed using GROMACS (v. 2021.4), parametrizing the systems according to previous studies.^[^ ^7^, ^8^ ^]^ Two independent 500 ns long replicas with 5 ns equilibration (coupling 2.5 ns of NVT to 2.5 ns of NPT) for each of the systems under analysis (amoxicillin‐PBP3, enantiomeric‐amoxicillin‐D‐PBP3, and amoxicillin‐D‐PBP3) were run starting from different initial velocities.

## Conflict of Interest

The authors declare no conflict of interest.

## Author Contributions

L.P. performed methodology, investigation, software, wrote the original draft and edited the final manuscript. G.G. performed wrote the original draft and edited, conceptualization; C.D. performed wrote the original draft and edited, conceptualization; L.D. performed methodology, formal analysis, investigation, wrote the original draft and edited, conceptualization, supervision.

## Data Availability

The data that support the findings of this study are available from the corresponding author upon reasonable request.
